# Overexpression of *MaTPD1A* impairs fruit and pollen development by modulating some regulators in *Musa itinerans*

**DOI:** 10.1186/s12870-020-02623-w

**Published:** 2020-08-31

**Authors:** Chunhua Hu, Ou Sheng, Tao Dong, Qiaosong Yang, Tongxin Dou, Chunyu Li, Weidi He, Huijun Gao, Ganjun Yi, Guiming Deng, Fangcheng Bi

**Affiliations:** 1 Institute of Fruit Tree Research, Guangdong Academy of Agricultural Sciences, Guangzhou, China; 2 Key Laboratory of South Subtropical Fruit Biology and Genetic Resource Utilization, Ministry of Agriculture, Guangzhou, China; 3 Guangdong Province Key Laboratory of Tropical and Subtropical Fruit Tree Research, Guangzhou, China

**Keywords:** Wild banana fruits develop, TPD1, Male sterile, *Musa itinerans*, Overexpression

## Abstract

**Background:**

Pollen formation and development is important for crop fertility and is a key factor for hybrid development. Previous reports have indicated that *Arabidopsis thaliana TAPETUM DETERMINANT1* (*AtTPD1*) and its rice (*Oryza sativa*) homolog, *OsTPD1*-like (*OsTDL1A*), are required for cell specialization and greatly affect pollen formation and development. Little is known about the role of the TPD1 homolog in banana pollen development.

**Results:**

Here, we report the identification and characterization of TPD1 homologs in diploid banana (*Musa itinerans*) and examine their role in pollen development by overexpressing the closest homolog, *MaTPD1A*. *MaTPD1A* exhibits high expression in stamen and localizes in the plasma membrane. *MaTPD1A*-overexpressing plants produce no pollen grains and smaller and seedless fruit compared to wild-type plants. Transcriptome analysis showed that in plant hormone, starch and sucrose metabolism, and linolenic acid metabolism-related pathways were affected by overexpression of *MaTPD1A*, and the expression of several key regulators, such as *PTC1* and *MYB80*, which are known to affect anther development, is affected in *MaTPD1A*-overexpressing lines.

**Conclusions:**

Our results indicate that MaTPD1A plays an important role in pollen formation and fruit development in diploid banana, possibly by affecting the expression of some key regulators of pollen development.

## Background

Pollen development, release, and pollination are vital for successful plant sexual reproduction and genetic diversity in the world. The development of hybrid lines with genetically modified (GM) pollen might group multiple important agronomic traits together and result in great hybrid vigor. In the anther of flowering plants, pollen formation results from the differentiation and interaction of reproductive cells (microsporocytes) with somatic anther wall cells (tapetum) [1]. In Arabidopsis and rice, an increasing number of genes that are involved in pollen development and formation have been determined through characterization of male sterile mutants in both species, and the key genes that control the process of pollen development in both species are relatively conserved [2–4].

In Arabidopsis, rice and Maize, a pair of protein-protein interactors that complex TAPETUM DETERMINANT1 (AtTPD1): EXCESS MICROSPOROCYTES1/EXTRA SPOROGENOUS CELLS (AtEMS1/EXS) in Arabidopsis [5–7], OsTDL1A: MULTIPLE SPOROCYTE1 (OsMSP1) in rice (*Oryza sativa*) [8–10], and MULTIPLE ARCHESPORIAL CELLS1): ZmMSP1(MULTIPLE SPOROCYTE1 (ZmMAC1) in maize (*Zea mays*) [11, 12] has been identified as a critical regulator for anther cell differentiation. Mutation of AtTPD1 results in complete male sterility due to missing pollen grains [5, 13], and analysis of *TPD1*-overexpressing plants showed that increased expression of *TPD1* activates cell division and results in increased cell number in the carpel through interacting with the EMS1/EXS receptor protein kinase [14].

Banana is an important fruit crop in tropical and subtropical areas. The wild banana trees are diploid, fruits develop if pollinated, and are fully seeded [15]. Parthenocarpy in some diploid bananas is proved to be involved in at least three dominant loci through crossing research between diploid seeded *Musa acuminata* (not parthenocarpic) and edible diploid bananas (parthenocarpic) [16]. Edible bananas are vegetatively parthenocarpic, and seedless fruits develop without pollination. The banana inflorescences are complex and contain female flowers, male flowers, and neutral flowers. The female flowers and male flowers are the main types of flowers in banana. Usually female flowers have an elongated trilocular ovary with three fused carpels and non-functional staminodes, and the style is surrounded by six petals. Male flowers have a smaller ovary, slender style and five stamens, and the stamens have long anthers that may or may not produce pollen. In the fertile wild banana, viable pollen is produced, but some pollen is produced by edible banana too [17]. The ovaries of female flowers develop into the fruit or finger, and clusters of banana fruit fingers are arranged into hands. The number of hands in the bunch depends on the number of female clusters in the inflorescence, and varies depending on the genotype and environmental conditions. In 1953, Fahn reported that in the Dwarf Cavendish banana plant the first flower is formed on the right side of the hand, and the remained flowers form in a zigzag pattern, back and forth between the top and the bottom rows [18]. In wild banana, there are five different patterns found for flower development. Two of them are similar to Fahn′s reported pattern. There is one pattern that flowers form from left to right side of the hand, and the order of flowers form is opposite to what Fahn described [19]. Wild banana plants are diploid, generally cross-pollinated, and have fertile seeds. Cultivated bananas are triploid, with a variable degree of parthenocarpy and sterility, and present a low number or complete absence of seeds [20]. Banana crossbreeding is the most important method to create new cultivars. In the past thirty years, many banana-planting countries launched a series of crossbreeding programs and released many useful cultivars [21, 22].

Pollen formation and vitality is an important factor for crossbreeding, but little is known about the molecular mechanism of pollen development and formation in banana. Here, we identified four homologs of TPD1 based on the released banana A genome. To investigate the role of MaTPD1A, the closest homolog of AtTPD1, we overexpressed *MaTPD1A* in the wild cultivar *M. itinerans*. Characterization of *MaTPD1A*-overexpressing (*MaTPD1A*-OE) plants indicated that TPD1A impairs fruit and pollen formation, and transcriptome analysis disclosed that many previously known anther development-related genes exhibit differential expression in the *MaTPD1A*-OE plant compared to WT. These data suggest that MaTPD1A modulates fruit and pollen development by affecting the expression of genes involved in these processes.

## Results

### Determination and characterization of the AtTPD1 homolog in the Banana a genome

**Fig. 1:**
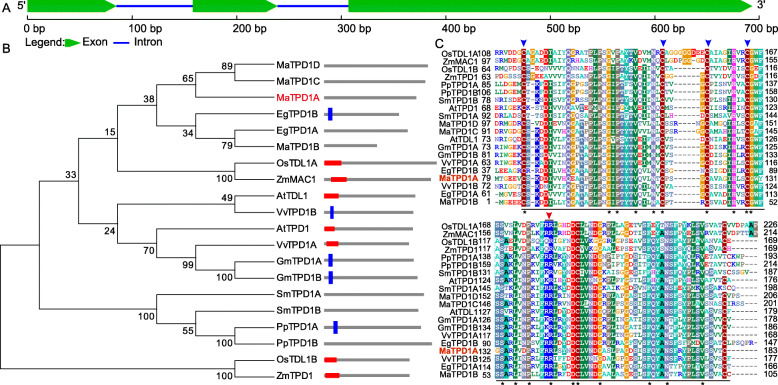
Sequence analysis of banana TPD1 homologs. **a** Schematic representation of the sequence organization of *MaTPD1A*. **b** Phylogenetic analysis of TPD1 and the related homologs. The amino acid sequences were aligned using Clustal Omega, and the tree was drawn with the neighbor-joining method using MEGA software 5.0 (http://megasoftware.net/). The architecture of each gene was obtained from SMART using the architecture analysis function. The red frame indicates a signal peptide; the blue frame indicates a transmembrane domain. EgTPD1A, PpTPD1B, SmTPD1A, SmTPD1B, MaTPD1A, MaTPD1C, and MaTPD1D had no matches in the SMART database. Detailed sequence information is as follows: AtTPD1 (AAR25553.1); AtTDL1 (ABF59206.1); EgTPD1A (XP_010936413.1); EgTPD1B (XP_019703285.1); GmTPD1A (XP_006581852.1); GmTPD1B (XP_014630314.1); OsTDL1A (BAG98429.1); OsTDL1B (BAH00567.1); PpTPD1A (XP_024360232.1); PpTPD1B (XP_024403645.1); SmTPD1A (XP_002981330.2); SmTPD1B (XP_002966893.2); VvTPD1A (XP_002280540.1); VvTPD1B (XP_010646260.1); ZmMAC1 (AEN03028.1); ZmTPD1 (ACG48634.1); MaTPD1A (Ma00_p03730.1); MaTPD1B (Ma08_p27040.1); MaTPD1C (Ma06_p07710.1); and MaTPD1D (Ma09_p01210.1). **c** CLUSTAL Omega alignment of TPD1 from various organisms showing the highly conserved region. At, *Arabidopsis thaliana*; Eg, *Elaeis guineensis*; Gm, *Glycine max*; Os*, Oryza sativa* Japonica Group; Pp, *Physcomitrella patens*; Sm*, Selaginella moellendorffii*; Vv, *Vitis vinifera*; Zm, *Zea mays*; Ma, *Musa acuminata*. The underlined region was the conserved C-terminal domain of TPD1 and its homologs; Blue arrow indicates the conserved cysteine residues that are essential for the normal function of AtTPD1; Red arrow representates the dibasic cleavage site of AtTPD1. Star indicates amino acids that are identical in all sequences

Using the Arabidopsis TPD1 (AAR25553) protein sequence as a query, a TBLASTN search was conducted against the banana A genome database. Four *TPD1* homolog genes were identified and named *MaTPD1A* (Fig. 1a), *MaTPD1B, MaTPD1C* and *MaTPD1D*, and the corresponding protein sequences were inferred Ma00_p03730.1, Ma08_p27040.1, Ma06_p07710.1 and Ma09_p01210.1, respectively. They have 183, 105, 201, and 206 amino acids, respectively. Moreover, the 9.05 and 10.05 isoelectric points of MaTPD1C and MaTPD1D are greater than the 5.74 and 5.15 PI of MaTPD1A and MaTPD1B, respectively. The blast search showed that MaTPD1A has conserved domains of AtTPD1 and shares 54% sequence identity and 75% sequence similarity with the Arabidopsis TPD1 protein (Fig. 1 and Table 1). MaTPD1B exhibits higher identity and similarity with AtTDL1 than the other three TPD1 homologs. Phylogenetic analysis shows that the four banana TPD1 homologs cluster into one class with two homologs from *Elaeis guineensis* and are more evolutionarily related to OsTDL1A and maize MULTIPLE ARCHESPORIAL CELLS 1 (ZmMAC1), which has been reported to have an important role in anther development [8, 10, 11] (Fig. 1b). All homologs of TPD1 have a highly conserved region at the C terminus (Fig. 1c). The blue arrows indicated four conserved cysteine residues, and the red arrow shows the dibasic cleavage site of AtTPD1. There is an apparent signal peptide or transmembrane domain in the N terminus of the TPD1 homolog in several species, but no signal peptide or transmembrane domain was detected in the four TPD1 homologs in banana (Fig. 1b). Subsequently, to determine the role of TPD1 homologs in banana, we selected the most conserved homolog of AtTPD1, MaTPD1A, for further analysis.

**Table 1:** Identity and similarity analysis of banana TPD1 homologs and TPD1 proteins of Arabidopsis

Identity / Similarity (%)	MaTPD1A (Ma00_p03730.1)	MaTPD1B (Ma08_p27040.1)	MaTPD1C (Ma06_p07710.1)	MaTPD1D (Ma09_p01210.1)
AtTPD1 (AAR25553.1)	54/75	54/73	46/67	46/66
AtTDL1 (ABF59206.1)	52/69	65/86	50/68	52/69

### Expression level of *MaTPD1A* in different tissues of banana

**Fig. 2:**
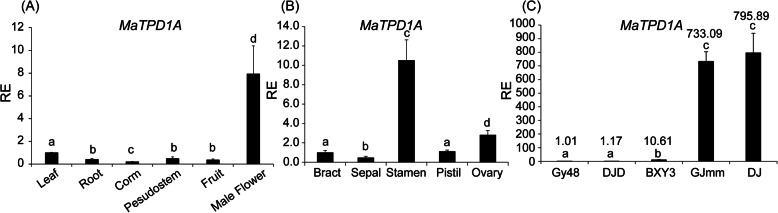
Expression of *MaTPD1A* in different tissues (**a**, **b**) and embryonic cell suspensions (**c**). GY48, DJD, BXY3, GJmm and DJ indicate the name of ECS induced from different banana varieties. *UBQ2* was used to normalize the expression data. Bars indicate standard deviation (SD). RE means relative expression level. The experiments are performed three times, and one representative experiment is presented. Letters indicate significantly different values using Fisher’s protected least significant difference, a post-hoc multiple t-test (*P* < 0.05)

The qRT-PCR analysis showed that *MaTPD1A* exhibits higher expression in male flower buds compared to that in other examined tissues (Fig. 2a). To further characterize the expression in different parts of male flowers, the male flower of wild-type plants was collected for qRT-PCR assays. The results indicate that MaTPD1A shows high expression in the stamen compared with that in other male flower tissues. (Fig. 2b). Additionally, we found that *MaTPD1A* shows high expression in newly established embryogenic cell suspensions (ECS) from two banana varieties and weak expression in non-embryogenic cell suspensions of three banana varieties (Fig. 2c). Moreover, the expression of *MaTPD1A* in the non-embryogenic callus of wild bananas is almost non-detectable (data not shown). These results indicate that MaTPD1A might play an important role in plant regeneration from the embryogenic callus.

### Subcellular localization of MaTPD1A in banana

**Fig. 3:**
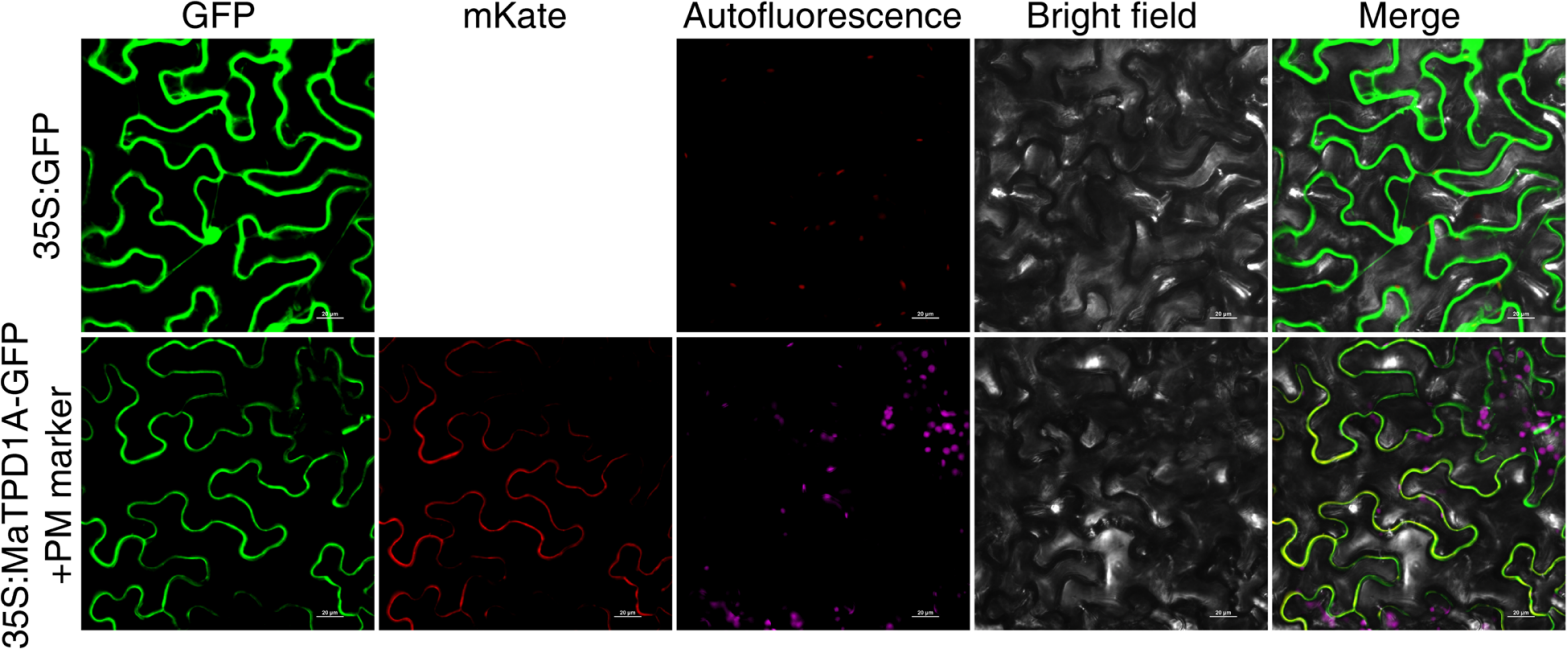
Subcellular localization analysis of MaTPD1A. The ORF of *MaTPD1A* was in frame with the GFP N-terminus, and the obtained construct and empty vector were transiently expressed in tobacco leaves by Agrobacterium-mediated transformation. The fluorescence signals were examined using a fluorescence microscope after 48 h of incubation. The mKate fluorescence signal indicates the plasma membrane. Overlay images show colocalization of GFP and mKate signals. Bar = 20 μm

To characterize the subcellular localization of MaTPD1A protein, we generated a construct in which the ORF of MaTPD1A was in frame with the N-terminus of GFP and under the control of the cauliflower mosaic virus 35S promoter (CaMV 35S). The localization was determined by transient expression of these constructs in tobacco epidermis cells by *Agrobacterium tumefaciens*-mediated transformation. As a control, GFP fluorescence was observed in the entire cell. When the MaTPD1A-GFP vector and plasma membrane marker vector were cotransformed into tobacco leaves, the MaTPD1A-GFP fusion protein colocalized with the plasma membrane marker, which suggests that MaTPD1A localizes exclusively to the plasma membrane (Fig. 3).

### Phenotypic characterization of MaTPD1A-OE lines

**Fig. 4:**
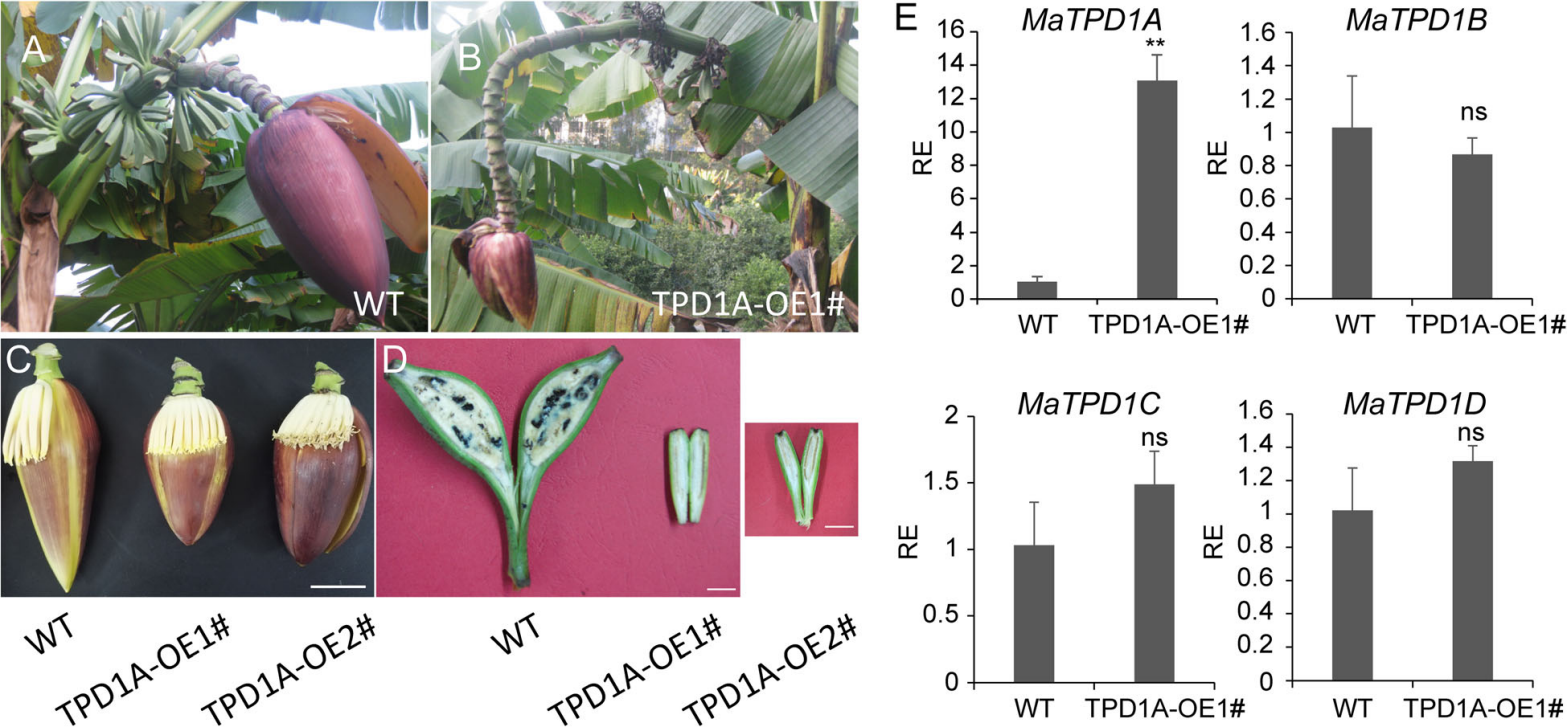
Characterization of fruit and buds in transgenic and wild-type plants. **a** The whole fruit bunch of wild-type plants; **b** The whole fruit bunch of transgenic plants; **c** Comparison of male flower buds from wild-type and transgenic plants. Bar = 5 cm; **d** Comparison of fruit fingers between wild-type and transgenic plants. Bar = 1 Cm. **e** Expression analysis of *TPD1* homologs in WT and *MaTPD1A*-OE line. The gene expression values are relative to that of the wild-type plant (set as 1). The experiments are performed three times, and one representative experiment is presented. RE indicates relative expression level. Significant differences were determined using Student’s t-test (**P < 0.01). Bars indicate standard deviation, ns means not significant

**Fig. 5:**
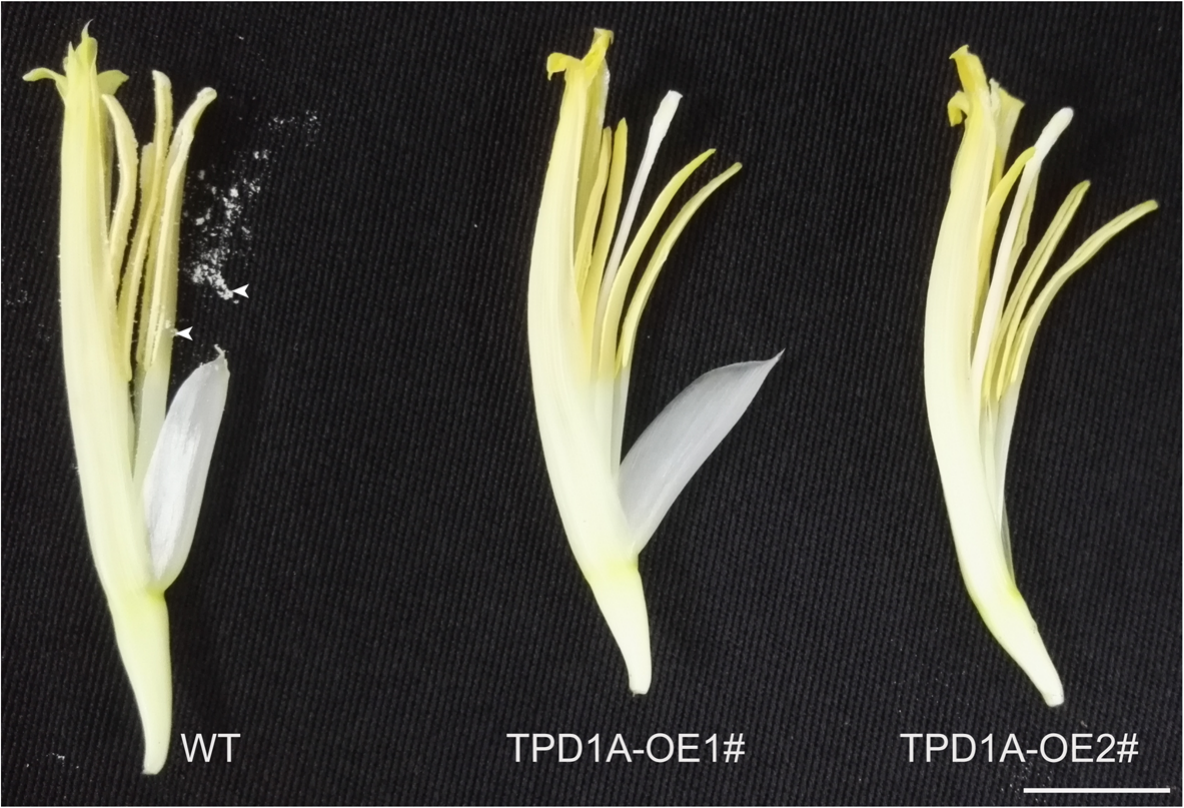
Characterization of male flowers in transgenic and wild-type plants. Arrows indicate pollen grains. Bar = 1 cm

To elucidate the role of *MaTPD1A* in banana, the cDNA of *MaTPD1A* was overexpressed in wild-type plants under the control of the constitutive cauliflower mosaic virus 35S promoter (35S). Constructs for overexpression were used to transform diploid banana embryogenic cell suspensions via Agrobacterium-mediated transformation. TPD1A homolog-overexpressing plantlets were transferred to soil and grown until anthesis. There are no apparent differences between wild-type and *MaTPD1A*-OE plants during the vegetative growth stage. In the reproductive growth stage, MaTPD1A-overexpressing plants can produce normal buds (Fig. 4a, b), but the male flowers of transgenic plants are a bit shorter than those of wild-type plants (Fig. 4c). Moreover, most fruit fingers wither and die in the late development stage in MaTPD1A-overexpressing plants, and the remaining fruit fingers appear small and seedless (Fig. 4b, d), suggesting an important role for MaTPD1A in fruit development. Importantly, the transgenic plants are male sterile and produce normal anthers that do not contain pollen grains (Fig. 5), indicating the critical function of MaTPD1A in pollen development in banana. We obtained 8 *MaTPD1A*-OE lines in total. The phenotype of all transgenic lines are the same, and similar to that of presented two *MaTPD1A*-OE lines (Fig. 4). Therefore, one representative MaTPD1A-OE line was selected for further analysis. The *MaTPD1A* transcript in *MaTPD1A*-OE1# line is approximately thirteen-fold higher than that of non-transformed plants, and the transcripts of the other three *TPD1* homologs are not affected by *MaTPD1A* overexpression (Fig. 4e).

### Transcriptome analysis between *MaTPD1A*-OE and wild-type lines

**Fig. 6:**
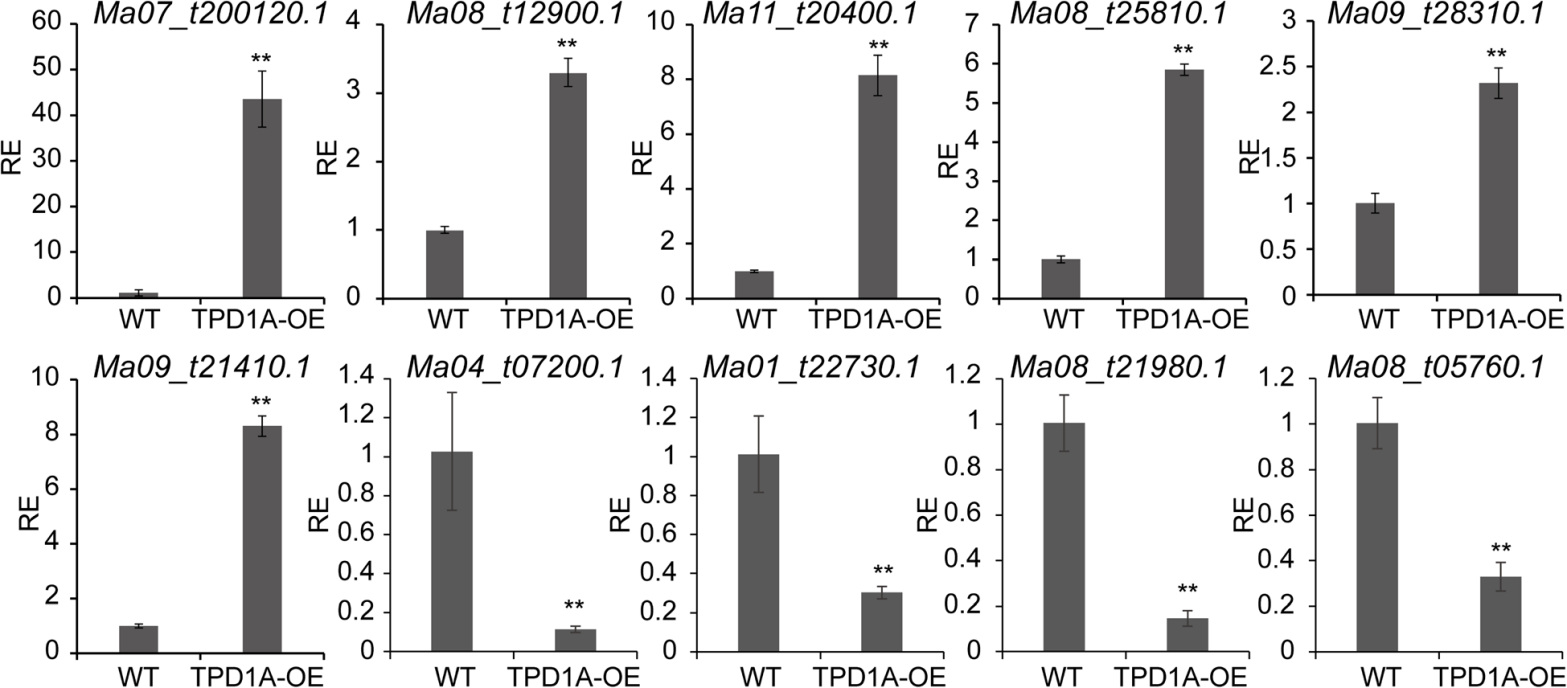
Expression level of ten selected genes involved in plant hormone pathways in wild-type and transgenic plants. The gene expression values are relative to that of the wild-type plant (set as 1). RE means relative expression level. *Ma07_t00120.1* (indole-3-acetic acid-amido synthetase *GH3.1*); *Ma08_t12900.1* (ethylene receptor 2-like); *Ma08_t25810.1* (jasmonic acid-amido synthetase *JAR1*); *Ma09_t21410.1* (auxin-induced protein 15A-like); *Ma09_t28310.1* (ETHYLENE INSENSITIVE 3-like 3 protein); *Ma11_t20400.1* (ethylene-responsive transcription factor 1B-like); *Ma08_t05760.1* (gibberellin receptor GID1C-like); *Ma08_t21980.1* (abscisic acid receptor PYL4-like); *Ma01_t22730.1* (brassinosteroid LRR receptor kinase-like); *Ma04_t07200.1* (probable indole-3-acetic acid-amido synthetase GH3.8). The experiments are performed three times, and representative data from one experiment is presented. Significant differences were determined using Student’s t-test (**P < 0.01). Bars represent standard deviation

To further characterize the effect of *MaTPD1A* overexpression on the entire transcriptome, RNA-Seq profiling was performed with male flower buds of wild-type and *TPD1A*-overexpressing plants (*MaTPD1A*-OE1#). The fragments per kilobase of transcript per million fragments mapped (FPKM) values were presented from three biological replicates for each sample (Additional File 2: Table S2). Base on a cut-off threshold of | Log2 (fold change) | > 1 and False Discovery Rate (FDR) < 0.01, a total of 3832 genes in the male flower were found to be differentially expressed in *MaTPD1A*-OE compared to WT (Additional File 3: Table S3). Among them, 2188 were upregulated and 1644 were downregulated (Additional File 3: Table S3). To test the reliability of transcriptome data, ten plant hormone signal transduction-related genes that are differentially expressed in *MaTPD1A*-OE plants were selected to examine in the reverse transcription-quantitative real time PCR (RT-qPCR) assay. The gene expression results of RT-qPCR exhibited similar trends with those of the transcriptome data with some variation in the magnitude (Fig. 6).

**Fig. 7:**
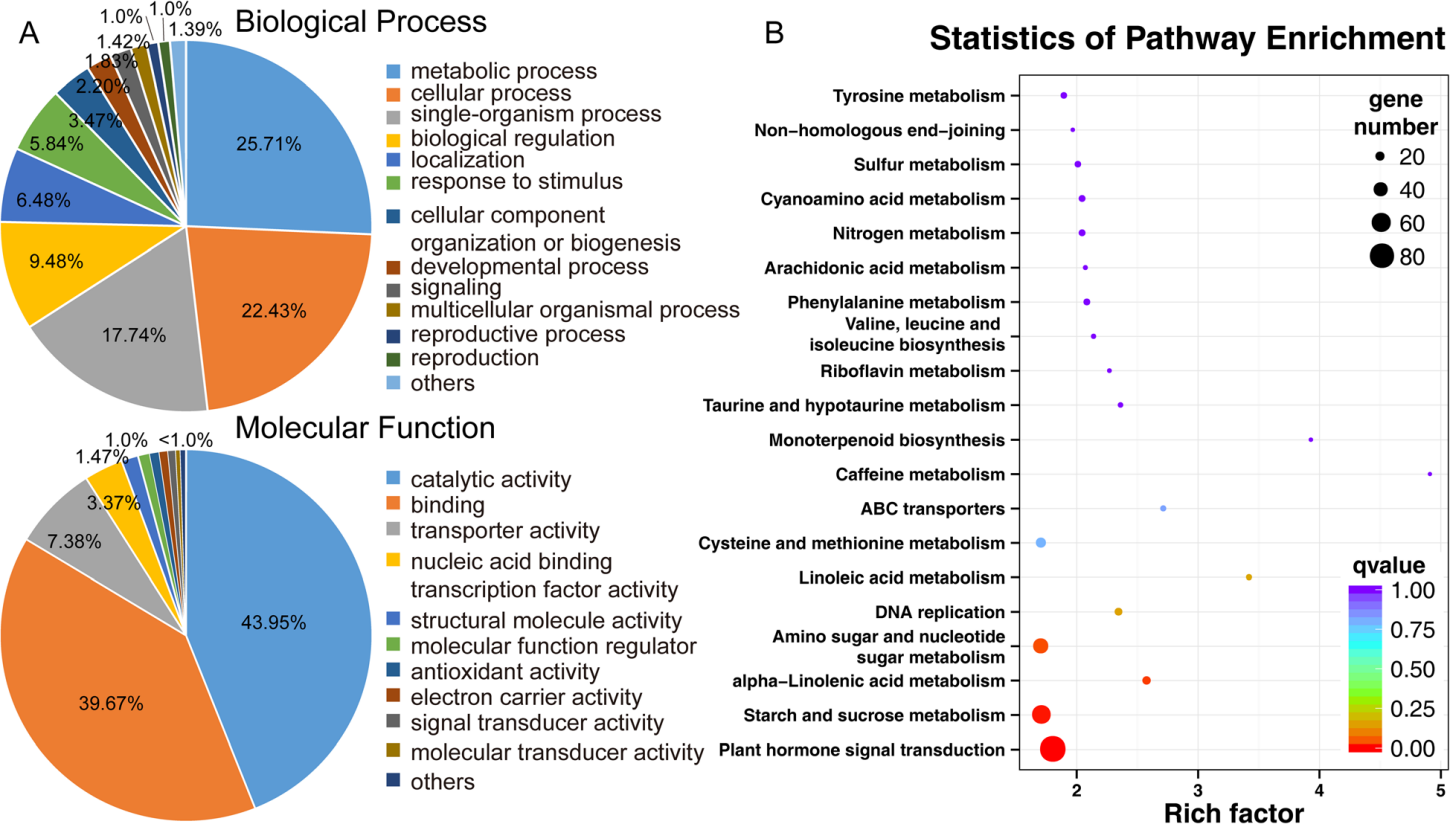
Functional categories and enriched pathways of the genes affected by MaTPD1A Overexpression. **a** The genes that differentially expressed in the MaTPD1A-OE plant were classified into several biological processes categories and molecular functional categories base on the Gene Ontology Consortium database and enrichment pathways; **b** Representation of the 20 most-enriched KEGG pathways. The significance of the enrichment factor is indicated with the coloring of the q-values. The target genes of enriched pathways are shown using a circle, and the gene number is proportional to the size of the circle. The Y-axis and the X-axis represent the name of the enrichment pathway and the enrichment factor, respectively

**Fig. 8:**
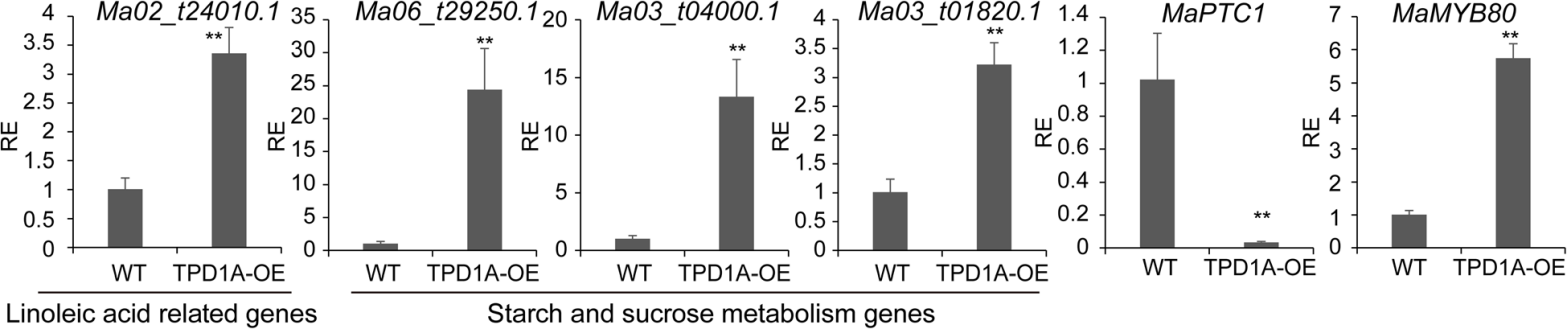
Expression level of genes involved in pollen development in anthers. The gene expression levels are relative to that of the wild-type plant (set as 1). RE means relative expression level. *Ma02_t24010.1* (acyl-coenzyme A oxidase 2); *Ma06_t29250.1* (beta-glucosidase 6-like isoform); *Ma03_t04000.1* (galacturonosyltransferase 4); *Ma03_t01820.1* (sucrose synthase 2); *Ma08_t23580.1* (PHD finger protein MALE STERILITY 1, *MaPTC1*); *Ma05_t24840.1* (transcription factor MYB80, *MaMYB80*). The experiments are performed three times, and one representative experiment is presented. Significant differences were determined using Student’s t-test (**P < 0.01). Bars indicate standard deviation

To assign these genes to different biological and molecular functional categories, Gene Ontology terms were determined and their enrichment was calculated. The functional annotation of each gene was retrieved from the Banana Genome database (*M. acuminata*, DH Pahang, Version 2). Regulation of metabolic process, cellular process, single-organism process, biological regulation, catalytic, binding and transporter activity related GO terms were enriched in differentially expressed genes in *TPD1A*-OE plants (Fig. 7a). Moreover, to determine the biological interpretation of differentially expressed genes, KEGG pathway analysis was performed. The results showed that 335 genes were assigned to the top 20 enriched pathways (Additional File 4: Table S4 and Fig. 7b). The first four significantly enriched terms were “Plant hormone signal transduction”, “Starch and sucrose metabolism”, “Amino sugar and nucleotide sugar metabolism”, and “alpha-linolenic acid metabolism” according to *P*-values (Additional File 4: Table S4). A total of 85, 58, and 17 genes related with plant hormone, starch and sucrose metabolism, and linolenic acid metabolism, respectively, were overrepresented (Additional File 5: Table S5, Additional File 6: Table S6, Additional File 7: Table S7); among those above, two-thirds of the genes were upregulated in *MaTPD1A*-OE plants. Additionally, 668 differentially expressed genes were assigned to the transcription factor category, suggesting that MaTPD1A overexpression affects the regulation of multiple transcription factors (Additional File 8: Table S8). We also confirmed the results through qRT-PCR analysis (Fig. 8). Notably, a series of genes that are homologs of key genes for anther and pollen development in rice or Arabidopsis were found to be differentially expressed in *MaTPD1A*-OE plants (Fig. 8, Table 2). PTC1 plays a vital role in tapetum formation and degradation. MYB80, UTD1 and ZmMADS2 transcription factors are critical regulators of pollen and anther development. MST8, UGP2, CP1 and SUT3 are required for sugar partitioning in Arabidopsis or rice. These results suggest that overexpression of MaTPD1A affects pollen development by modulating transcription of anther or pollen development-associated genes.

**Table 2:** Expression data of previously reported pollen and anther development-related genes in MaTPD1A-OE male flowers

Locus ID of homolog	Name	Putative Function	Log2FC	FDR
*Ma08_t23580.1*	*PTC1*	PHD finger protein PERSISTENT TAPETAL CELL 1	−4.92	7.07E-56
*Ma05_t09200.1*	*UTD1*	Transcription factor ABORTED MICROSPORES	1.38	9.19E-08
*Ma05_t24840.1*	*MYB80*	MYB family transcription factor	2.47	2.12E-27
*Ma08_t01350.1*	*MST8*	Sugar carrier protein C	4.61	6.04E-73
*Ma03_t28150.1*	*UGP2*	UTP--glucose-1-phosphate uridylyltransferase	1.09	0.00110607
*Ma06_t12130.1*	*SUT3*	Sucrose transport protein SUT1	2.61	1.42E-08

## Discussion

The development of pollen is a tightly regulated process determined by a series of specific functional factors [3, 23]. Impairment of gene function might affect anther or pollen formation or result in pollen abortion and male sterility. Understanding the molecular and biochemical mechanisms of anther and pollen development-related genes are very important for both basic and applied research for banana cross breeding. In this study, we first identified the AtTPD1 homologs in banana and examined the effect of overexpressing the *TPD1* homolog in banana pollen development. The results establish a foundation for further understanding the role of MaTPD1A in anther development and pollen formation.

In Arabidopsis, TPD1 and EMS1 are vital regulators of anther cell differentiation, and mutation of either gene results in excess microsporocytes and no tapetal cells [5, 24]. *AtTPD1* is expressed in the leaves, young seedlings and flower buds, whereas its rice homologs *OsTDL1A* and *OsTDL1B* are predominantly expressed in the root and flowers [4, 8]. Moreover, at the different stages of flower bud development, *TPD1* expression in both Arabidopsis and rice is dynamic. There was another *TPD1*-like gene named *AtTDL1* in Arabidopsis, but its role in pollen development has been unknown until now. A recent report indicated that among the two TPD1 homologs of rice, only OsTDL1A is coexpressed with MSP1 in ovules and binds MSP1 for limiting sporocyte numbers, though they exhibit similar expression patterns in different tissues [8]. This report suggested that they might have different roles in flower development. In the banana genome, four TPD1 homologs were identified with the AtTPD1 protein sequence through TBLASTp queries. The banana *MaTPD1A* transcript was mainly identified in male flower buds and stamens (Fig. 2), which is consistent with its role in pollen development. Moreover, our results indicated that the expression level is closely related to the regenerative capacity of different ECSs. It is necessary to further determine the role of MaTPD1A in regeneration of the ECS.

In Arabidopsis, mutation analysis of TPD1 revealed that its N-terminal signal peptide is essential for its normal function in anther cell differentiation [6], and AtTPD1 can be processed into a small protein by cleaving at KR cleavage site. AtTPD1 localizes in trafficking vesicles under normal conditions, and TPD1 can be secreted to the plasma membrane in the presence of EMS1 [6]. Two TPD1 homologs of rice also have N-terminal signals (Fig. 1b), suggesting that TPD1 homologs of rice might function with a similar mechanism. TPD1 determines the fate of functional tapetal cells, whereas mutation or disruption of the signal peptide affects its plasma membrane localization regardless of the presence of EMS1 [6]. In banana, the N-terminal signal peptide and KR cleavage site are missing in all four TPD1 homologs (Fig. 1b, c). It means that MaTPD1 might be not a secreted protein and function with intact protein. In the MaTPD1 homologs, the KR cleavage site was instead of ‘RR’, which is similar to OsTDL1A and ZmMAC1 (Fig. 1c). The MAC1 protein is an intact protein that does not seem to be processed except removal of the putative signal peptide [11]. In Arabidopsis, some cysteine-rich proteins AtRALF contain RR dibasic sites (RRXL), and can be processed by a plant subtilisin-like serine protease [25]. Further experiments are required for verifying if MaTPD1 can be cleaving at the ‘RR’ site. In addition, it is possible that some cleavage sites may exist in other places of these proteins. The subcellular localization analysis of MaTPD1A determined its localization in the plasma membrane. But no N-terminal signal peptide was found using SMART. One possible explanation is that there are potential signal peptides in other position of MaTPD1A, another possible explanation is that MaTPD1A might functions at the plasma membrane through interacting with plasma membrane-localized partners such as the EMS1 homolog in Arabidopsis [6]. It would be worth examing it in the future. The above results indicate that MaTPD1A in pollen formation might function through different mechanisms than AtTPD1 and OsTDL1A.

In Arabidopsis, the *TPD1* mutant plants have no pollen grains and produce small siliques without seeds [4], and overexpression of *TPD1* results in increased epidermal cells and wide siliques [13]. *MaTPD1A*-overexpressing plants produce no pollen grains in male flowers, and female flowers of transgenic plants develop into small fruits without seeds (Figs. 4, 5). One possible explanation is that TPD1 in diploid banana has different mechanisms than that in Arabidopsis in pollen formation and development. Additionally, our transcriptome analysis indicates that a series of genes involved in pollen and anther development were differentially expressed between *MaTPD1A*-overexpressing plants and wild-type plants (Table 2). In our *MaTPD1A*-overexpressing plants, the transcript of the *PTC1* ortholog was significantly suppressed (Fig. 8, Table 2). A previous report showed that the *PTC1*-mutant rice plant had smaller anthers and no viable pollen grains [26], and a similar phenotype was observed in barley plants with the ortholog silenced [27], similar to our *MaTPD1*-overexpressing plants, implying that downregulation of the *PTC1* ortholog in banana might contribute to the male sterile phenotype. Moreover, expression of the upstream transcription factor *AtMYB80* and *OsUTD1* orthologs is also affected by overexpression of *MaTPD1A* (Fig. 8, Table 2). AtMYB80 and OsUTD1 have been reported to be key regulators of pollen development in Arabidopsis and rice, respectively [28, 29]. The sugar partition-related proteins MST8, UGP2, and SUT3 also participate in rice pollen development [30, 31]. Additionally, we found some starch and sucrose metabolism- and linoleic acid-related genes that were differentially expressed in *MaTPD1A*-OE banana male flowers (Fig. 8, Additional File 6: Table S6, Additional File 7: Table S7). Research on many male sterile lines has shown that carbohydrates are involved in anther and pollen development as nutrient and signaling factors [32, 33]. In cucumber, downregulation of the sucrose transporter *CsSUT1* results in male sterility by affecting carbohydrate supply [34]. Linoleic acid is also a critical factor that affects pollen development and fertility [35]. Hormone signaling, such as the auxin signaling pathway, is also closely involved in anther and pollen development [36]. It has been reported that increased expression of the auxin biosynthesis gene *FvYU6* affects flower development and male fertility in strawberry [37], and male sterility due to high temperature in Arabidopsis can be reversed by auxin application [38]. Our qRT-PCR results indicate that overexpression of *MaTPD1A* greatly affects the transcription of several auxin signaling-related genes (*Ma09_t21410.1, Ma07_t00120.1, Ma04_t07200.1*) in diploid banana. In some parthenocarpic bananas, The developmental program controls a series of key genes to support the formation of embryos without fertilization and male sterility, which was reminiscent of male sterility induced by MaTPD1A overexpression. A similar program could be activated in *MaTPD1A*-OE plants. Therefore, we believed that in these two genotypes plants there are some overlapping genes that for controlling similar phenotype. Further research to compare the expression profile of *MaTPD1A*-OE plants with that of parthenocarpic bananas would be beneficial for understanding the underlying molecular mechanism. In addition, the expression profile of female and neutral flowers is also helpful for us to disclose the role of MaTPD1A on pollen and fruit development.

Taken together, our results indicate that overexpression of *MaTPD1A* in diploid banana affects the expression level of numerous genes that related to pollen development, and cause male sterile phenotype. The underlying mechanism is still obscure. Mutation of *MaTPD1A* with CRISPR/Cas9 mediated gene editing or RNAi of *MaTPD1A* will provide more information for its role of *MaTPD1A* in pollen formation and development. This study provides a basis for further studies to confirm its roles in anther and pollen development and for manipulation of banana fertility in the future.

## Conclusions

In this research, we examined the effect of overexpressing *MaTPD1A* on pollen and fruit development in diploid banana. MaTPD1A is the most closest homolog of TPD1 in banana and localized to the plasma membrane. Overexpression of *MaTPD1A* in diploid banana results in the missing of pollen grains in male flowers, and producing small and seedless fruit. Further transcriptome analysis indicated numerous genes previously reported to be involved in pollen development were up- or down-regulated in *MaTPD1A*-overexpressing plants. Therefore, our results revealed that MaTPD1A modulates pollen formation through regulating the expression of some relative regulators, which provides a basis for further studies to confirm its roles in anther and pollen development and for manipulation of banana fertility in the future.

## Methods

### Plant materials and growth conditions

*M. itinerans*, known also as Yunnan banana, is wide distribution across subtropical China, and can be found in moist ravines to mountainous areas in China (https://banana-genome-hub.southgreen.fr/organism/Musa/Itinerans). The *M. itinerans* that we used was collected from Jinji Mountain, Pingxiang City, Guangxi Province in China, propagated and maintained in national banana germplasm repository, Institute of Fruit Tree Research, Guangdong Academy of Agricultural Sciences (IFTR, GAAS, Guangzhou, China). The sample collection got permission from the local administration department. After identification by Dr. Ou Sheng who is working on banana germplasm collection and identification for many years, the multiple bud clumps of *M. itinerans* have also been deposited in public in vitro repository for clonal banana germplasm in IFTR, GAAS, and the deposited number was NYBGZXJ01404. Its genome sequence has been determined and released in 2016 [39]. The embryogenic cell suspensions (ECSs) used for transformation were induced from male flowers of *M. itinerans* maintained in the national banana germplasm repository. For banana plantlets cultivation potted banana plantlets were grown up to the five true leaves stage in a greenhouse before they were transplanted into the experimental field for further cultivation under the following condition: natural photoperiod (11/13 h) with a temperature of 18–30 °C under 60–80% relative humidity. The field trial in plastic house obtained permission from the biosecurity regulators in Guangdong Academy of Agricultural Sciences (GAAS, Guangzhou, China) to conduct this trial.

### Vector construction and transformation

The full-length *TPD1A* homolog open reading frame (ORF) was amplified with the following primer set: forward (5′-ATGGCCTGCTTCCACGGTTTG-3′) and reverse (5′-TCAGCAAGTGACGGAGGAGAC-3′). The reaction condition was as follows: 94 °C for 3 min; 32 cycles of 94 °C for 15 s, 55 °C for 30 s and 72 °C for 30 s, and the PCR products were separated and extracted in 1.0% (w/v) agarose gels. This ORF was introduced into the pMD18-T vector (D101A, TaKaRa, Dalian, China) for sequencing. The correct MaTPD1A ORF fragment was collected and subcloned into the pBI121 vector after double digestion with *Sac* I (TaKaRa, Dalian, China) and *Xba* I (TaKaRa, Dalian, China). The pBI121:*MaTPD1A* plasmid was digested with *Eco*R I (TaKaRa, Dalian, China) and *Hin*d III and cloned into the pCAMBIA1301 vector (CAMBIA). The obtained pCMABIA1301:*MaTPD1A* construct was transformed into *A. tumefaciens* strain EHA105 for transformation with a previously described method [40]. Briefly, the embryogenic cell suspensions (ECSs) of *M. itinerans* were cocultured with *A. tumefaciens* for 24 h at 26 °C with gentle shaking in the dark. The ECSs were collected by removing the supernatant, washed twice with sterile water, and cultured in proliferation medium supplemented with 25 mg/L hygromycin B and 200 mg/L cefotaxime at 26 °C with shaking at 110 rpm in the dark. Next, the ECSs were cultivated in regenerated medium and sub-cultured every 3 weeks for 3 times total until resistant mature somatic embryos appeared. The regenerated seedlings were confirmed with PCR amplification of the hygromycin resistance gene, and positive banana plants were multiplied using meristems of in vitro plantlets. The rooted plantlets were transplanted into the greenhouse and cultivated for further study.

### Subcellular localization

The full-length ORF of *MaTPD1A* was cloned in frame with GFP in the pCMABIA1300-GFP vector. The pCMABIA1300-GFP vector was modified from binary pCMABIA1300 vector, and the CaMV 35S promoter, GFP, RBS terminator were introduced into pCAMBIA1300 with *Eco*R Iand *Sac* I, *Pst* I, *Pst* Iand *Hin*d III, respectively. The obtained construct was transformed into *A. tumefaciens* strain EHA105 for further use. The transient expression of MaTPD1A:GFP was performed as previously described [41]. Images were obtained using a confocal laser-scanning microscope (Nikon C2-ER, Tokyo, Japan).

### RNA extraction and qRT-PCR analysis

Total RNA was extracted from different samples using a plant RNA isolation kit (Column Plant RNAOUT 2.0, TIANDZ, China) according to the manufacturer’s instructions. The different tissue samples including leaf, root, corm, pseudostem, fruit and male flower were collected from the banana plant at 30 days after flowering. For banana inflorescence the female flowers appear first, the distal portion of the inflorescence elongates and produces clusters of male flowers. When the female flowers develop into fruit, we collected male flowers and immature fruit samples at the same time. The cDNA template was synthesized from 1 μg total RNA using PrimeScript RT reagent kit with gDNA Eraser (TAKARA, Dalian, China). For qRT-PCR assays, 5 μL of TB Green Premix Ex Taq II (TAKARA), 4.0 μL of diluted cDNA, 0.8 μL of gene-specific primers, and 0.2 μL of ROX Reference Dye were mixed together and the reaction was run in a StepOne Plus (ABI) with the following program: 30 s at 95 °C followed by 40 cycles of 95 °C for 5 s and 60 °C for 30 s. The *UBQ2* (HQ853254.1) expression level was selected as an internal control [42]. The relative expression of target genes was evaluated using 2-△△Ct method [43] relative to the internal control. The primers used in this study are listed in Additional File 1: Table S1. All experiments are repeated three times, and one representative experiment is presented.

### Transcriptome analysis

The male flower bud of wild-type and *TPD1A*-OE1# line plants were collected at about 90 days after flower and ground into fine powder using liquid nitrogen. There were three replicates for each sample. Total RNA was extracted using the methods described above. RNA quality and concentration were evaluated by a NanoDrop 2000 UV-Vis Spectrophotometer (Thermo Fisher Scientific, Wilmington, DE, USA), and the integrity of the RNA was determined with an Agilent 2100 Bioanalyzer (Agilent Technologies, Palo Alto, USA). The paired-end library was prepared using a TruSeq RNA Sample Preparation kit (Illumina, San Diego, USA), and the resulting sequencing libraries were used for RNA-seq analysis by Biomarker Technologies Corporation (Beijing, China) with the HiSeq × 10 platform.

## Supplementary information

**Additional file 1: Table S1.** Primers used in this study.

**Additional file 2: Table S2.** Full genes that examined in transcriptome analysis.

**Additional file 3: Table S3.** Differentially expressed genes in *MaTPD1A*-OE plants.

**Additional file 4: Table S4.** top 20 enriched pathways.

**Additional file 5: Table S5.** Differentially expressed genes involved in plant hormone signal transduction.

**Additional file 6: Table S6.** Differentially expressed genes involved starch and sucrose metabolism.

**Additional file 7: Table S7.** Differentially expressed genes involved linolenic acid metabolism.

**Additional file 8: Table S8.** Differentially expressed transcription factors in *MaTPD1A*-OE plants.

## Data Availability

The transcriptomic data are available in the repository of NCBI with the accession number PRJNA606623 (https://www.ncbi.nlm.nih.gov/sra/?term=PRJNA606623). The data sets supporting the results of this article are included within the article and its additional files.

## Abbreviations

TPD1: TAPETUM DETERMINANT1
TDL1: TPD1 like 1
PTC1: PERSISTENT TAPETAL CELL 1
GM: Genetically modified
MAC1: MULTIPLE ARCHESPORIAL CELLS1
EMS1/EXS: EXCESS MICROSPOROCYTES1/EXTRA SPOROGENOUS CELLS
MSP1: MULTIPLE SPOROCYTE1
ORF: Open reading frame
GFP: Green fluorescent protein
OE: Overexpression
FDR: False discovery rate
